# Foxtail mosaic virus-induced gene silencing (VIGS) in switchgrass (*Panicum virgatum* L.)

**DOI:** 10.1186/s13007-022-00903-0

**Published:** 2022-05-30

**Authors:** Kira Tiedge, Janessa Destremps, Janet Solano-Sanchez, Magda Lisette Arce-Rodriguez, Philipp Zerbe

**Affiliations:** 1 Department of Plant Biology, University of California, Davis, USA; 2 Groningen Institute for Evolutionary Life Sciences, University of Groningen, Groningen, The Netherlands

**Keywords:** Switchgrass (*Panicum virgatum*), Virus-induced gene silencing (VIGS), Bioenergy crops, Foxtail mosaic virus (FoMV)

## Abstract

**Background:**

Although the genome for the allotetraploid bioenergy crop switchgrass (*Panicum virgatum*) has been established, limitations in mutant resources have hampered *in planta* gene function studies toward crop optimization. Virus-induced gene silencing (VIGS) is a versatile technique for transient genetic studies. Here we report the implementation of foxtail mosaic virus (FoMV)-mediated gene silencing in switchgrass in above- and below-ground tissues and at different developmental stages.

**Results:**

The study demonstrated that leaf rub-inoculation is a suitable method for systemic gene silencing in switchgrass. For all three visual marker genes, *Magnesium chelatase subunit D* (*ChlD*) and *I* (*ChlI*) as well as *phytoene desaturase* (*PDS*), phenotypic changes were observed in leaves, albeit at different intensities. Gene silencing efficiency was verified by RT-PCR for all tested genes. Notably, systemic gene silencing was also observed in roots, although silencing efficiency was stronger in leaves (~ 63–94%) as compared to roots (~ 48–78%). Plants at a later developmental stage were moderately less amenable to VIGS than younger plants, but also less perturbed by the viral infection.

**Conclusions:**

Using FoMV-mediated VIGS could be achieved in switchgrass leaves and roots, providing an alternative approach for studying gene functions and physiological traits in this important bioenergy crop.

**Supplementary Information:**

The online version contains supplementary material available at 10.1186/s13007-022-00903-0.

## Background

The perennial grass switchgrass (*Panicum virgatum* L.) is a major species of the North American tallgrass prairies and of agroeconomic importance as a C4 lignocellulosic biofuel feedstock crop. Its high net energy efficiency and environmental hardiness allow biofuel production on marginal lands with minimal agricultural input [1–3]. Two major switchgrass ecotypes, lowland and upland, are distinguished that feature large variation in habitat adaptation, morphological characteristics and ploidy levels [4]. Lowland ecotypes are predominantly tetraploid, while upland ecotypes mostly contain octoploid genomes [2, 4, 5]. Recent sequencing of the allotetraploid genome of the lowland ecotype Alamo AP13 has enabled the investigation of gene functions and corresponding physiological traits, which lays the foundation for crop optimization through increasing switchgrasses’ environmental resilience [2]. However, the genetic diversity and high self-incompatibility of switchgrass ecotypes have limited genetic studies and the development of mutant resources [6, 7]. Transformation protocols and methods for CRISPR/Cas9- and RNAi-mediated gene editing in switchgrass have been reported [8–13]. For example, the pANIC vector collection has been successfully employed for the stable downregulation of lignin biosynthetic genes in transgenic embryogenic callus cultures [14, 15] and have been used in transgenic RNAi studies to analyze and regulate cellulose and lignin biosynthesis in switchgrass [10, 16]. These and other efforts have further been integrated to reduce cell wall recalcitrance in transgenic switchgrass lines to enable more cost-efficient lignocellulosic biofuel production [8, 11].

Virus-induced gene silencing (VIGS) has been established as an alternate tool for transient gene function studies [17, 18]. VIGS takes advantage of evolutionarily conserved antiviral defense mechanisms via post-transcriptional gene silencing of viral RNA [19]. In general, integration of a target gene into a viral vector system is used to trigger the degradation of specific plant genes of interest [20, 21]. In the past decade, VIGS has been successfully used to investigate the function of genes with relevance to plant development and stress defenses in several monocot crops, including rice (*Oryza sativa*) [22, 23], maize (*Zea mays*) [24–30], wheat (*Triticum aestivum*)[30], and barley (*Hordeum vulgare*) [31, 32]. A range of different VIGS vectors is now available that offer a broad host range and high silencing efficiency and duration and have proven useful for gene function studies associated with stress resistance in monocot plants [26, 27, 31, 33, 34]. For example, foxtail mosaic virus (FoMV) has been shown to successfully silence gene expression in a range of monocot species, including barley, wheat, foxtail millet (*Setaria italica*, and maize [26, 31], thus providing a promising tool for gene silencing in switchgrass. The independence of VIGS-mediated gene silencing with respect to stable transformation protocols and defined genetic backgrounds further provides a versatile resource for transient gene function studies that impact pathways and traits that vary across tissues and plant developmental stages. For example, barley stripe mosaic virus (BSMV)-enabled VIGS was used to affect gene silencing in wheat leaves, roots and grain tissue as well as different developmental stages [35, 36]. Similarly, key enzymes involved in the production of defensive, specialized glycoalkaloid metabolites were confirmed via a combination of VIGS, quantitative trait loci (QTL) analysis and metabolomics in tomato (*Solanum sp.*) [37].

In this study, we selected three widely used marker genes, namely *Magnesium chelatase subunit D* (*ChlD*) and *subunit I* (*ChlI*) involved in chlorophyll biosynthesis [38, 39] and *phytoene desaturase* (*PDS*) functioning in carotenoid metabolism [40] to establish FoMV-mediated VIGS in different switchgrass tissues and developmental stages.

## Methods

### Gene identification and construct generation

Given the allotetraploid genome of *Panicum virgatum*, orthologues of the targeted marker genes *PDS*, *ChlD*, and *ChlI* were identified via annotation search and a BLAST search with the *ZmPDS* (GRMZM2G410515) sequence [26] of the switchgrass reference genome (Alamo AP13, v5; [2]). Nucleotide sequences were aligned in Geneious Prime (Biomatters, USA) and highly conserved 200–400 bp nucleotide fragments for each gene were selected for VIGS. These fragments were amplified from switchgrass cDNA with forward and reverse oligonucleotides adding *Xba*I and *Pac*I restriction sites, respectively (Additional file 1: Table S3). Amplicons were then ligated into the infectious FoMV vector [26]. The final constructs were transformed into *E. coli* DH5α (New England Biolabs, USA) and sequence verified prior to experimental use.

### Plant cultivation

Seeds of switchgrass (*Panicum virgatum* var. Alamo AP13) were obtained from the U.S. Department of Agriculture Germplasm Resources Information Network (accession no. PI 422006). Seeds of *N. benthamiana* were kindly provided by Dr. Katayoon Dehesh (University of California, Riverside, USA). For both *N. benthamiana* and switchgrass, approximately 6–8 seeds were planted in 3 × 3 inch pots in growth chambers at 23 °C, 16 h light/8 h dark cycles, and watered at weekly intervals. After two weeks, successfully germinated seedlings were transferred to individual 6 × 6 inch pots and grown under the same conditions until infiltration. The temperature was lowered to 21 °C after *Agrobacterium tumefaciens* GV3101 infiltration.

### Agroinfiltration of N. benthamiana

Transformation of *A. tumefaciens* with the generated constructs was performed as reported before [41]. Here, competent *A. tumefaciens* GV3101 cells were transformed via heat shock and tested for the presence of the targeted inserts. Transformed *A. tumefaciens* were streaked on LB agar plates containing 50 µg/ml kanamycin and rifampicin and incubated at 28 °C for 2 days. A single colony of *A. tumefaciens* was used to inoculate 5 ml LB liquid medium with the aforementioned antibiotics and incubated at 28 °C for 24 h at 180 rpm, after which 5 ml LB culture was used to inoculate 30 ml of LB medium with the antibiotics and subsequent incubation at the above conditions. Transformed cells were pelleted by centrifugation at 3000 rpm for 15 min and resuspended twice in 30 ml of 10 mM MgCl_2_. Fresh infiltration solution was used to resuspend the cells, followed by incubation at 28 °C for 3–4 h at 200 rpm at which time the culture was diluted to an OD_600nm_ of 1 with infiltration medium (prepare fresh before infiltration: 10 mM MgCl2, 10 mM 2-(N-morpholino)ethanesulfonic acid (MES), and 150 μm acetosyringone). Finally, 4.6 µl of Silwet L-77 (Bioworld, USA) was added with further incubation for 10 min. For agroinfiltration, the abaxial side of two leaves was infiltrated per plant with a 1 ml needleless syringe and placed in a growth chamber at 21 °C without light for 24 h and then with light for 3 weeks post-infiltration.

To confirm the systemic virus infection of the *N. benthamiana* plants, leaf and root tissue was harvested for RT-PCR, sequence verification, and stored at − 80 °C until further analysis. Subsequently, the tissue was ground to a fine powder with autoclaved and bleached mortars and pestles for RNA extractions (Monarch Total RNA Miniprep Kit, New England Biolabs), cDNA synthesis (SuperScript™ III First-Strand Synthesis System, Invitrogen) and PCR (GoTaq^®^ DNA Polymerase, Promega). The amplification protocol was as follows: initial denaturation at 95 °C for 2 min; followed by 35 cycles of denaturation at 95 °C for 30 s, annealing at 60 °C for 45 s, and extension at 72 °C for 60 s; completed by final elongation. Oligonucleotide sequences are given in Additional file 1: Table S3. Amplicons were analyzed via sanger sequencing (Quintarabio, USA).

### Rub-inoculation of switchgrass

To prepare the virus-containing *N. benthamiana* sap, 4 g of leaf material displaying viral symptoms (as confirmed by RT-PCR) were collected. Rub-inoculation of *P*. *virgatum* seedlings that reached either elongation stage E1 or elongation stage E3 followed an established protocol ([26]. *N. benthamiana* leaf tissue was ground using a mortar and pestle in 16 ml of KP inoculation buffer with 500 mg of silicon carbide powder (600 grit; Beta Diamond Products, USA) added. For rub-inoculation, sterile cotton swabs were dipped into the sap and used to inoculate the second and third leaf from the apex (Additional file 1: Fig. S1). The rub-inoculation should be forceful enough to mediate the infection while preventing extensive leaf damage (a squeaking sound from stripping the wax layer of the leaves should be audible). Inoculated seedlings were maintained in growth chambers at 21 °C or 22 °C without light for 24 h and then with a 16 h photoperiod. Symptoms of infection were monitored 1–3 weeks post-inoculation. After a period of four weeks, inoculated seedlings were harvested for analysis at a range of timepoints to assess construct stability and silencing efficiency.

### Confirmation of systemic virus infection

Confirmation of virus infection in *P. virgatum* was carried out four weeks post-inoculation. Root and leaf tissue was harvested, immediately flash frozen in liquid nitrogen, and stored at − 80 °C. Plant tissue was ground in bleached and twice-autoclaved mortar and pestles followed by RNA extraction using the Monarch Total RNA Miniprep Kit (New England Biolabs, USA). RT-PCR experiments were performed to detect the FoMV vector and the *ChlD*, *ChlI*, and *PDS* target genes using gene-specific oligonucleotides (Additional file 1: Table S3). Wild-type RNA was used as a negative control. GoTaq^®^ Green Master Mix (Promega, USA) was used to conduct RT-PCR amplification. The amplification protocol was as follows: initial denaturation at 95 °C for 2 min; followed by 35 cycles of denaturation at 95 °C for 30 s, annealing at 60 °C for 45 s, and extension at 72 °C for 60 s; completed by final elongation. The oligonucleotides used can be found in Additional file 1: Table S3. Amplicons were analyzed via Sanger sequencing (Quintarabio, USA).

### *Validation of silencing efficiency *via* qPCR*

For qPCR analysis, *18SrRNA* (18SrRNA1) and *actin* (ACT12) were used as reference genes based on previous studies in *P*. *virgatum* [42, 43], as well as efficiency tests using different template concentrations (oligonucleotide sequences are given in Additional file 1: Table S3). Primers for target genes were designed outside the insert regions and tested for product specificity and an efficiency of 100 ± 5%. QPRC reactions were performed in a 10 μl volume with 5 μl iTaq Universal SYBR Green Supermix (Bio-Rad Laboratories, USA), 2 × 0.2 μl of oligonucleotides, and 2 μl of the respective cDNA with the following parameters: initial denaturation at 95 °C for 30 s; 40 cycles of denaturation at 95 °C for 10 s, annealing at 60 °C for 10 s, and extension at 72 °C for 20 s. After quality analysis of melting curves, Bio-Rad CFX Manager 3.1 was used to calculate the C_t_ values. Data analysis was performed using the ΔΔC_t_ method as described previously [44, 45]. Gene expression values were calculated on the basis of six biological replicates and calculated as the normalized relative quantity (NRQ) compared to the wild type. Statistical significance for gene expression was analyzed using a heteroscedastic Student’s *t*-Test with a two-tailed distribution (*p*-value < 0.05).

## Results

### Systemic infection of switchgrass plants

**Fig.1:**
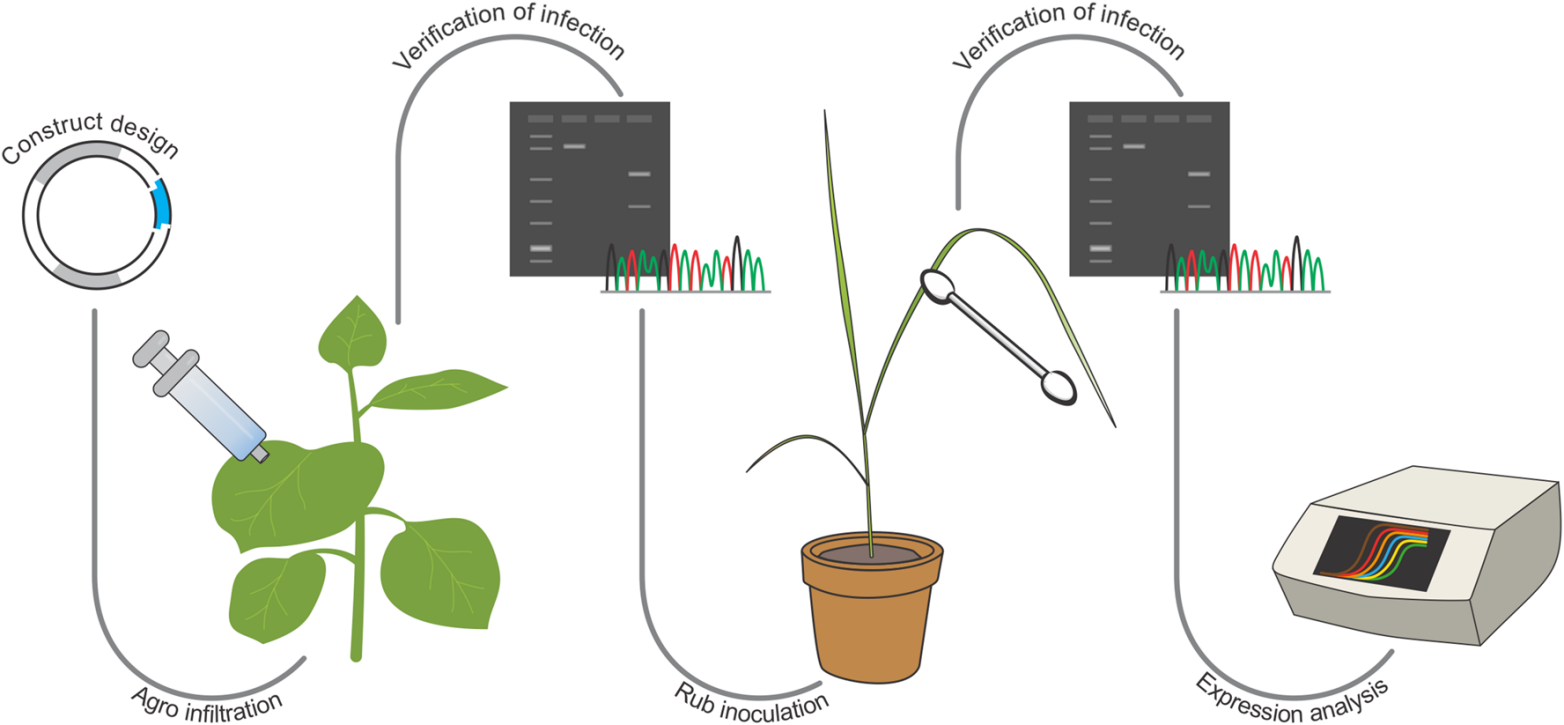
Workflow for VIGS in switchgrass. Fragments of the target genes inserted into the foxtail mosaic vector (FoMV) system are first transfected into *Nicotiana benthamiana* leaves for multiplication of the virus. After PCR verification of successful transformation, leaf sap is extracted and used to mechanically rub-inoculate switchgrass leaves, followed by PCR-verification of transformants and gene expression analysis of target genes via RT-PCR

FoMV-mediated VIGS has been successfully used to silence genes in monocot crops, including maize and *Setaria viridis* [26, 27, 31]. In addition, the suitability of mechanically inoculating plants by rubbing sap of infected *N. benthamiana* plants onto the leaves has been demonstrated in maize and other species [26]. Adopting this strategy to achieve gene silencing in switchgrass, three marker genes, switchgrass (*P. virgatum* var Alamo AP13) *magnesium chelatase* subunits D (*ChlD*) and I (*ChlI*) as well as *phytoene desaturase* (*PDS*) were chosen for gene silencing. The function of magnesium chelatase and phytoene desaturase in chlorophyll and carotenoid metabolism, respectively, results in a bleaching phenotype that enables visual screening for successful gene silencing [38–40]. Mining of the switchgrass genome (*P. virgatum* Alamo AP13 *v5.1*) revealed two highly conserved gene copies of each *ChlD* and *ChlI* with 98% and 89% protein sequence similarity, respectively. For putative *PDS* genes, six less conserved copies were identified which share an average sequence similarity of 26% (Additional file 1: Table S1). To achieve an efficient silencing of these identified genes, conserved regions for each gene group were identified (Additional file 1: Table S2). Using the resulting FoMV:ChlD, FoMV:ChlI and FoMV:PDS constructs, three-week-old *N. benthamiana* plants were agroinfiltrated either with the empty FoMV vector or one of the constructs for multiplication of the virus (Fig. 1).

**Fig. 2:**
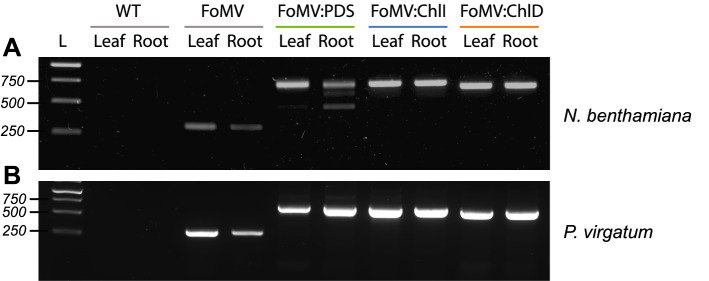
RT-PCR analysis of construct presence and integrity in *N. benthamiana* and *P. virgatum*. RT-PCR assays of systemic *N. benthamiana* (**A**) and *P. virgatum* (**B**) leaves and roots verifying the presence of the constructs FoMV:PDS, FoMV:ChlI or FoMV:ChlD as compared to wild type plants (no virus) and FoMV transfection only. Expected amplicon size is 314 bp for FoMV, 410 bp for FoMV:PDS, 424 bp for FoMV:ChlI, and 369 bp for FoMV:ChlD. Oligonucleotide sequences are listed in Additional file 1: Table S3

Two weeks after infiltration, gene-specific RT-PCR analysis verified the presence of the viral load in systemic leaves and roots for all infected tobacco plants (Fig. 2A). It should be noted that in case of the FoMV:PDS construct, RT-PCR indicated a deletion within the FoMV:PDS construct in new leaves and roots of some plants (Fig. 2A), a phenomenon observed frequently in VIGS studies [27]. Additionally, RT-PCR products were sequenced to verify successful infection of *N. benthamiana* with the correct constructs. Having established sufficient virus production in *N. benthamiana*, the second and third leaf from the apex of 3-week-old *P. virgatum* seedlings were inoculated using rub-inoculation with *N. benthamiana* sap containing the respective constructs or FoMV empty vector as a negative control. RT-PCR analysis showed successful systemic infection in newly emerged leaves of all inoculated plants (*n* = 45), and in 28% of the tested plants a systemic infection was also observed in roots. In other monocot crops like maize a systemic FoMV infection of the seedlings established itself around 14 days post-inoculation (dpi) [27]. Considering the relative slower growth rate of switchgrass as compared to these crops, we tested different intervals between inoculation and sampling to achieve a suitable balance between stability of the constructs and efficient silencing, which became established at approximately 28–32 dpi (Additional file 1: Fig. S3). Stability of the construct and of the silencing effect was assessed via RT-PCR and *q*RT-PCR, respectively. In most cases, the silencing effect remained stable up to 48 dpi, but for FoMV:ChlD leaves as well as roots the efficiency decreased drastically after 44 dpi (Additional file 1: Fig. S3).

### Phenotypic changes

Infection of *N. benthamiana* with the FoMV:PDS, FoMV:ChlI or FoMV:ChlD constructs did not result in a substantial bleaching phenotype in *N. benthamiana* plants, likely due to the sequence differences between the switchgrass and *N. benthamiana* genes for *PDS*, *ChlI* and *ChlD,* which share 80% or less similarity at the nucleotide level (Additional file 1: Figs. S4–S6). However, some *N. benthamiana* leaves displayed a mosaic pattern after successful infiltration, which was also visible in plants containing only the empty FoMV vector and hence is presumably caused by the FoMV infection (Additional file 1: Fig. S2).

**Fig. 3:**
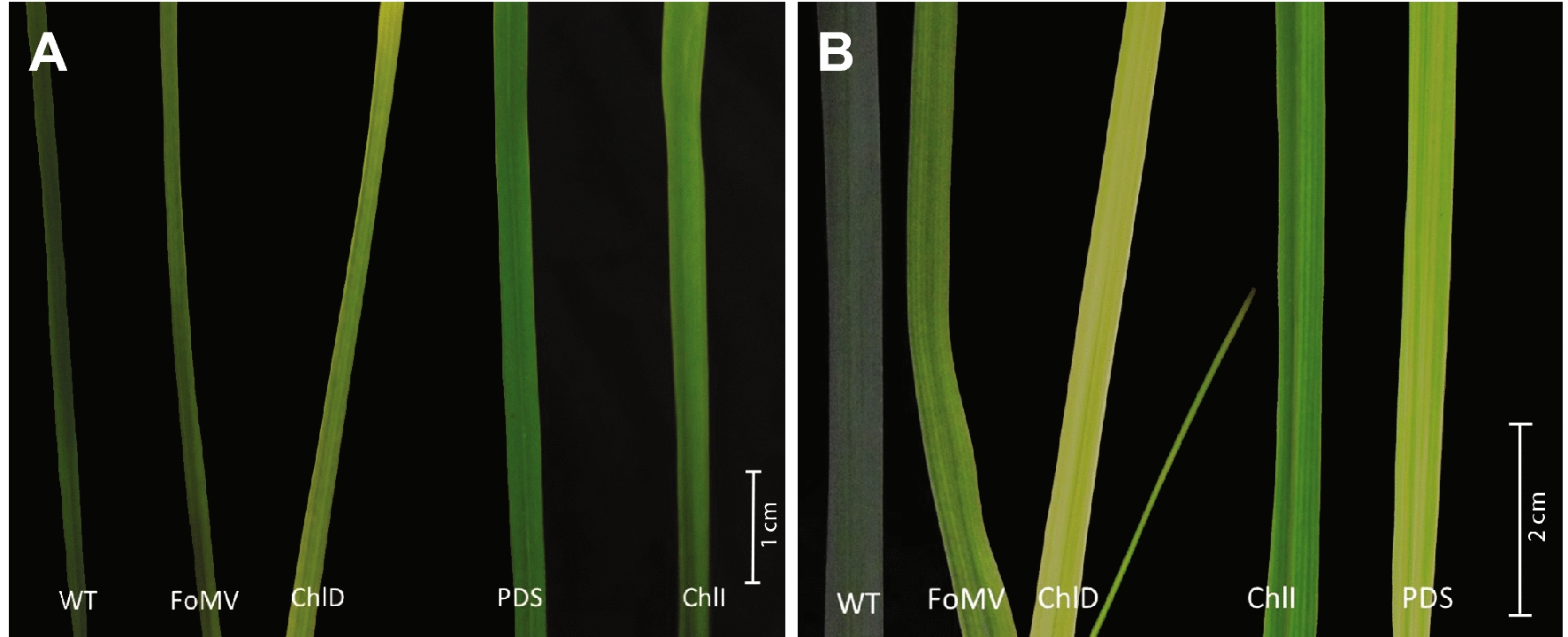
Phenotypic differences among older and younger switchgrass leaves 4 weeks post-inoculation (**A**) in younger plants that were infected at the E1 stage, (**B**) in older plants that were infected at the E3 stage. *WT:* wildtype/untreated plants; *FoMV:* empty vector control; *ChlD:* plants infected with FoMV:ChlD; *ChlI:* plants infected with FoMV:ChlI; *PDS:* plants infected with FoMV:PDS

After rub-inoculation with *N. benthamiana* leaf sap, switchgrass plants exhibited a range of phenotypes that varied between developmental stages and the construct used (Fig. 3, Additional file 1: Fig. S7). The two tested developmental stages included switchgrass plants that were inoculated at elongation stage 1 (E1) and plants that were inoculated at elongation stage 3 (E3). For plants that were inoculated at the E1 stage, leaves of untreated wild type plants (WT) showed a dark green pigmentation (Fig. 3A). Among the targeted genes, *ChlD* silencing resulted in the strongest bleaching phenotype in leaves, followed by *ChlI* and *PDS* (Fig. 3A). The leaves of plants infected with the empty FoMV vector showed less discoloration compared to plants infected with FoMV:ChlD, FoMV:ChlI, or FoMV:PDS (Fig. 3A). In older plants that were inoculated at the E3 stage similar, yet more pronounced, visual phenotypic patterns were observed (Fig. 3B). All FoMV:ChlD, FoMV:ChlI or FoMV:PDS plants showed phenotypic differences compared to the control plants. The strongest bleaching effect was detected in FoMV:ChlD plants, whereas the FoMV:ChlI plants only showed moderate bleaching comparable to the empty vector control plants (Fig. 3B). Not all plants in this study showed a bleaching phenotype and of those plants that showed a phenotype, mostly the older leaves were impacted. The total number of plants exhibiting a bleaching phenotype was 5–13% higher in plants that were inoculated at a later developmental stage and 20% higher in the empty vector control plants (Additional file 1: Table S4).

### Tissue-specific gene silencing

**Fig. 4:**
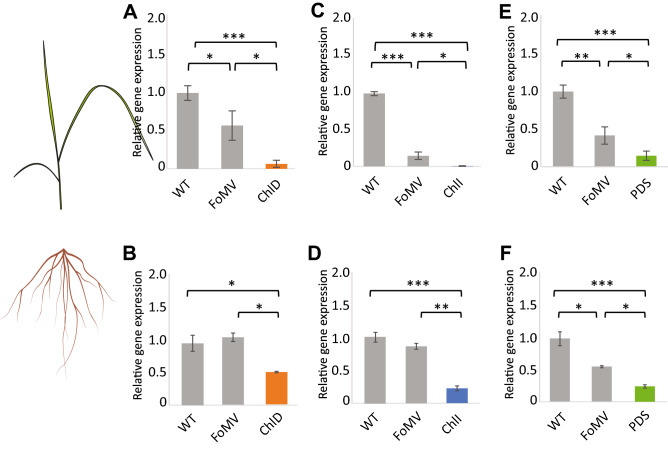
Gene expression levels 28 days after inoculation in leaves and roots for all three constructs and controls of switchgrass that was infected at the E1 stage. Expression of ChlD in control and FoMV:ChlD (**A**) leaves and (**B**) roots; expression of ChlI in control and FoMV:ChlI (**C**) leaves and (**D**) roots; expression of PDS in control and FoMV:PDS (**E**) leaves and (**F**) roots; *n* = 6 per biological sample group; no inoculated leaves but only newly emerged leaves were sampled for gene expression analysis; *p* ≦ 0.05 *; *p* ≦ 0.01 **; *p* ≦ 0.001***. WT, untreated wild type plants

Having detected systemic viral infection in both switchgrass leaves and roots (Fig. 2), we next assessed the gene silencing efficiency of our constructs 28 days after inoculation using RT-qPCR with leaf and root samples of switchgrass plants at the E1 stage and compared the relative gene expression to untreated wild type and empty vector control plants. ​For all three tested genes, gene expression was significantly decreased in infected seedlings as compared to the empty FoMV vector and wild type plants (Fig. 4). In leaf tissue, the average (*n* = 35) gene silencing efficiency was 87% for FoMV:ChlD, 90% for FoMV:ChlI, and 74.5% for FoMV:PDS as compared to wild type plants (Fig. 4A, C, E). Expectedly, FoMV vector control samples also were significantly different from wild type plants, reflecting the detrimental effect of viral infection on young leaves. In root tissue, a strong silencing effect was also observed, albeit less pronounced as compared to leaves. The average gene silencing efficiency was measured at 48% for FoMV:ChlD, 78% for FoMV:ChlI, and 76% for FoMV:PDS. In contrast to leaf tissue, the effect of viral infection itself on gene expression in roots was not significant, with the exception of FoMV:PDS (Fig. 4F).

### Gene silencing efficiency at different growth stages

**Fig. 5:**
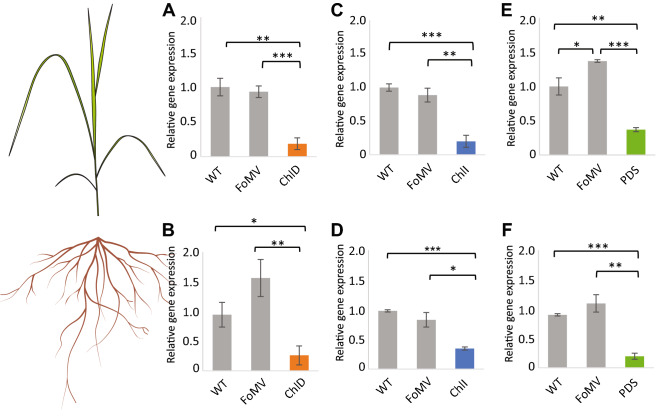
Gene expression levels 28 days after inoculation in leaves and roots for all three constructs and controls of switchgrass plants that were infected at the E3 stage. Expression of ChlD in control and FoMV:ChlD (**A**) leaves and (**B**) roots; expression of ChlI in control and FoMV:ChlI (**C**) leaves and (**D**) roots; expression of PDS in control and FoMV:PDS (**E**) leaves and (**F**) roots; *n* = 6 per biological sample group; no inoculated leaves but only newly emerged leaves were sampled for gene expression analysis; *p* ≦ 0.05 *; *p* ≦ 0.01 **; *p* ≦ 0.001***. WT, untreated wild type plants

To next evaluate gene silencing efficiency in older switchgrass, plants at the E3 developmental stage were analyzed via RT-qPCR and compared to plants at the E1 stage. In contrast to E1 plants, with the exception of FoMV:PDS leaves, gene expression in older E3 plants was not significantly impacted by the viral infection, resulting in similar expression levels between wild type and FoMV vector controls (Fig. 5). In some cases, a slight increase of transcript was detected in the empty vector control plants (Fig. 5B, E, F). For FoMV:ChlD a reduction in gene expression of 81% in leaves and 73% in roots was calculated, whereas FoMV:ChlI showed a decrease of 80% in leaves and 65% in roots as compared to 63% in leaves and 78% in roots for FoMV:PDS (Fig. 5). Thus, E3 seedlings displayed moderately stronger gene silencing in leaves as compared to the roots, similar to the younger plants. Collectively, these findings show that gene silencing had a comparable efficiency in leaves and a higher efficiency in roots of E3 plants compared to E1 plants, while the younger E1 seedlings showed more pronounced detrimental impact by the viral infection.

## Discussion

Advances in omics technologies have dramatically increased the availability of plant genomes and computational gene function annotations. Forward and reverse genetic studies are critical to empirically validate functional predictions *in planta*, yet remain challenging for many crops. Although CRISPR/Cas9 and RNAi protocols have been successfully employed in switchgrass [8, 10, 11, 14, 46, 47] , the complex allotetraploid genome and largely outcrossing nature of switchgrass complicate genetic studies. In this study, we used the FoMV vector and mechanical rub-inoculation, previously established in maize, sorghum, and wheat [26, 48, 49] to achieve VIGS in switchgrass. Using this approach, systemic infection was achieved in approximately one quarter (~ 28%) of the tested 3- or 8-week-old switchgrass seedlings. This infection efficiency is consistent with other monocot species, where plant susceptibility to viral infection has been shown to be impacted by other factors such as genotype/cultivar, vector chassis, construct design, and growth conditions [48, 49]. The relevance of optimizing construct design is exemplified by the instability of the FoMV:PDS construct observed in this study. We observed what presumably are deletion products of the construct in select samples. This might point to a possible issue of construct stability as described earlier in this study. This phenomenon has been a long-standing impediment for this technology, and efforts have already been made to improve the insert stability, for example through modified viral vectors and adjustment of environmental conditions [24, 50, 51]. In addition, the presence of gene copies for each of the *ChlD*, *ChlI* and *PDS* marker genes in the allotetraploid switchgrass genome necessitated the careful design of target gene fragments to avoid limitations in VIGS efficiency due to gene functional redundancies.

To visualize the knock-down, we used three different reporter genes which are commonly employed in gene editing studies and which are resulting in a visible phenotype in case of a successful silencing event. *Magnesium chelatase subunits D* (*ChlD*) and *I* (*ChlI*) act as regulators in the biosynthetic pathway of chlorophyll. Therefore, a loss of function in *ChlD* and *ChlI* inhibits chlorophyll biosynthesis resulting in a bleaching phenotype [38]. In addition, the targeted endogenous *phytoene desaturase* (*PDS*) encodes an enzyme catalyzing the first step in the carotenoid biosynthetic pathway. Carotenoids act as photoprotectants, therefore the knock-down of the transcript results in white leaves due to a photobleaching effect [40]. At the phenotypic level, the presence of mild bleaching of switchgrass leaves upon infection with the FoMV vector is similar to observations for FoMV infection in other monocot species [27]. For example, gene silencing of *PDS* or *ChlH* did not result in leaf bleaching in sorghum [48]. Although a stronger bleaching phenotype could be observed for all constructs targeted in this study, for FoMV:ChlD, FoMV:ChlI and FoMV:PDS, the phenotypic changes showed a large degree of variation. It is possible that different levels of bleaching symptoms caused by the FoMV infection alone mask the gene silencing effects of the target gene [48]. In addition, since two or more gene copies were targeted for each marker gene, the amount of functional protein produced may differ between infected switchgrass plants. It also cannot be excluded that in addition to the photosensitization-preventing mechanisms of carotenoids switchgrass can employ alternative mechanisms/pathways to provide photoprotection as shown in other crops [52]. The phenotypic variation among even successfully infected plants highlights the importance of verifying gene silencing at the molecular level. Indeed, RT-PCR analysis verified the successful silencing of all target genes with efficiencies of 74–90% in leaves and 48–78% in roots of 3-week-old switchgrass seedlings. Since experiments with different time periods between inoculation and sampling have shown that efficiencies can be impacted by extending the inoculation period, it is advisable to adjust the experimental design according to the used switchgrass variety, growth conditions and gene constructs.

The systemic gene silencing in both switchgrass leaf and root tissue can enable the analysis of metabolic processes and pathways that function in a spatiotemporal manner. Prominently, this includes defensive specialized metabolite pathways that are often expressed in specific tissues, at specific developmental stages and in response to environmental stimuli as demonstrated for monocot crops including switchgrass, rice, and maize [53–58]. Although *ChlD*, *ChlI*, and *PDS* gene silencing was slightly less efficient on average in switchgrass roots than in leaves, the detected gene silencing levels in roots were comparable to other crops including BSMV-mediated VIGS in wheat [35], bean pod mottle virus (BPMV)-enabled silencing in peas (*Pisum sativum*) [59], and cassava (*Manihot esculenta*) using the cassava gemini virus [60].

The majority of VIGS studies focuses on early developmental stages to take advantage of the higher gene silencing efficiency in younger plants, even though younger plants are also more susceptible to the viral infection as shown in *Arabidopsis* and other plant species [17, 61–63]. However, expanding VIGS studies across developmental stages can enable the investigation of biological processes and pathways that function only at later developmental stages. Hence, we here tested the efficiency of VIGS in switchgrass seedlings infected at two growth stages using 3- and 8-week-old seedlings at the E1 and E3 stage, respectively. 3-week-old plants exhibited a less pronounced bleaching phenotype and were more susceptible to viral infection as reflected in a lower relative gene expression in FoMV empty vector control plants. By contrast, accompanying a lower virus susceptibility, VIGS efficiency in leaves of 8-week-old plants was reduced by only 6–12%. In the 8-week-old seedlings, in some cases an increase of gene expression was detected in the empty vector control plants, which has been observed in other plant species before [64–66]. Surprisingly, with the exception of FoMV:ChlI, gene silencing efficiency was increased in roots of older switchgrass seedlings. This result shows that the efficiency of VIGS can be substantially impacted by the developmental stage of the plants used for silencing of specific genes. This effect has already been observed in other crops such as opium poppy [67] and gerbera [68]. A plausible explanation for this variation in VIGS efficiency includes differences in the activity of the host RNAi and of constitutive promotors including the Cauliflower mosaic virus 35S promoter of the FoMV vector at different developmental stages, especially in monocot plants [69, 70]. For example, expression levels driven by the same promoter were strong and constitutive in some studied lines, but in other lines expression levels were only increased in metabolically more active tissues [70]. While a deeper understanding of the underlying mechanisms would require extensive future studies as more constructs are being tested in switchgrass, these results indicate the suitability of older switchgrass plants for tissue-specific VIGS studies.

## Conclusions

VIGS offers a powerful tool for transient gene function studies, especially for species where plant transformation, regeneration, and genetic backgrounds present experimental challenges. Although variable phenotypic results and the presence of multiple gene copies in the switchgrass genome will likely require protocol and construct optimization of individual target genes, this study highlights the suitability of FoMV-mediated VIGS via rub-inoculation in switchgrass. Efficient systemic gene silencing in leaves and roots of switchgrass plants at different growth stages provides a resource for the *in planta* analysis of spatiotemporal genes and pathways in this bioenergy crop.

## Supplementary Information

**Additional file 1: Table S1.** Sequence information of marker genes revealed by genome mining of the AP13 switchgrass genome v5 (phytozome-next.jgi.doe.gov). **Table S2.** Sequences and constructs used for this study. **Table S3.** Oligonucleotide sequences used for this study .**Table S4.** Phenotypic evaluations for the different developmental stages and FoMV constructs. **Figure S1.** Rub-inoculation of switchgrass leaves at the elongation stage 3 (E3) with a cotton swab and the inoculum based on infected tobacco leaves. **Figure S2.** Tobacco (*N. benthamiana*) leaves displaying characteristic symptoms of successful infection with the foxtail mosaic virus (FoMV). **Figure S3.** Time course experiments to test efficiency and stability of gene silencing for PDS, ChlI, and ChlD are following the same methods as the other experiments except for a varied period between rub-inoculation and sampling (dpi) of switchgrass plants (*n*=6). **Figure S4.** Alignment of the amino acid sequences of Mg-Chelatase D Subunits (ChlD) from tobacco and switchgrass (Identity = 81.86%). **Figure S5.** Alignment of the amino acid sequences of Mg-Chelatase I Subunits (ChlI) from tobacco and switchgrass (Identity = 75.94%). **Figure S6.** Alignment of the amino acid sequences of phytoene desaturase (PDS) from tobacco and switchgrass (Identity = 78.11%). **Figure S7.** Photos of switchgrass plants that were infected at different developmental stages (either E1 or E3).

## Data Availability

Sequence data generated and analyzed in this study are included in this article and its Additional file information. The switchgrass reference genome v5 used for this study is available at phytozome-next.jgi.doe.gov [2].
