# Supplementary material for: Foxtail mosaic virus-induced gene silencing (VIGS) in switchgrass (Panicum virgatum L.)
Source: Plant Methods. 2022 May 30;18:71. doi: 10.1186/s13007-022-00903-0 (PMC9150325; doi:10.1186/s13007-022-00903-0)
Supplement: Supplementary file 1 — Additional file 1: Table S1. Sequence information of marker genes revealed by genome mining of the AP13 switchgrass genome v5 (phytozome-next.jgi.doe.gov). Table S2. Sequences and constructs used for this study. Table S3. Oligonucleotide sequences used for this study .Table S4. Phenotypic evaluations for the different developmental stages and FoMV constructs. Figure S1. Rub-inoculation of switchgrass leaves at the elongation stage 3 (E3) with a cotton swab and the inoculum based on infected tobacco leaves. Figure S2. Tobacco (N. benthamiana) leaves displaying characteristic symptoms of successful infection with the foxtail mosaic virus (FoMV). Figure S3. Time course experiments to test efficiency and stability of gene silencing for PDS, ChlI, and ChlD are following the same methods as the other experiments except for a varied period between rub-inoculation and sampling (dpi) of switchgrass plants (n=6). Figure S4. Alignment of the amino acid sequences of Mg-Chelatase D Subunits (ChlD) from tobacco and switchgrass (Identity = 81.86%). Figure S5. Alignment of the amino acid sequences of Mg-Chelatase I Subunits (ChlI) from tobacco and switchgrass (Identity = 75.94%). Figure S6. Alignment of the amino acid sequences of phytoene desaturase (PDS) from tobacco and switchgrass (Identity = 78.11%). Figure S7. Photos of switchgrass plants that were infected at different developmental stages (either E1 or E3). [file 13007_2022_903_MOESM1_ESM.pdf]

Additional Files: Foxtail mosaic virus-induced gene silencing (VIGS) in switchgrass (*Panicum virgatum* L.)

Supplementary Table S1: Sequence information of marker genes revealed by genome mining of the AP13 switchgrass genome v5 (phytozome-next.jgi.doe.gov).

**Enzyme name**	**Gene IDs**
ChlD	Pavir.9KG399531
	Pavir.9NG061903
ChlI	Pavir.3KG040848
	Pavir.3NG028600
PDS	Pavir.2KG542500
	Pavir.3NG267700
	Pavir.7KG288200
	Pavir.7NG283000
	Pavir.9KG650900
	Pavir.9NG463900

Supplementary Table S2: Sequences and constructs used for this study

Sequence insert for FoMV:ChlD construct (368 bp)
CACAAGATCAACAGCAAGAACAACCACCCCAACCACCACCCCCACCCCCTCCAGAAAACCAAGATTCTTCAGAAGACCAAGATGAGGAGGAAGAGGACGATCAAGAGGATGACGAGGAAGAAAATGAACAACAAGATCAGCAGATACCTGAGGAGTTCATATTTGATGCTGAAGGTGGTTTAGTAGATGATAAGCTCCTTTTCTTTGCTCAGCAAGCGCAAAGACGGCGAGGAAAAGCTGGGCGAGCAAAGAATGTCATATTCTCAGAAGATAGGGGTCGATACATAAAGCCTATGCTTCCAAAGGGTCCAGTAAGGAGATTAGCTGTTGATGCCACGCTTAGAGCAGCTGCACCGTACCAAAAACTG
Sequence insert for FoMV:ChlI construct (412 bp)
CCAACAGGGGGATACTCTACGTCGACGAGGTCAACCTGCTGGACGACCACCTCGTCGATGTGCTGCTGGATTCTGCCGCATCGGGGTGGAACACGGTGGAGAGGGAGGGTATCTCCATATCCCACCCTGCCCGATTCATCCTTATCGGCTCTGGTAACCCGGAGGAAGGGGAGCTCCGGCCGCAGCTGCTGGACAGGTTTGGGATGCACGCGCAGGTCGGTACGGTCAGGGACGCTGAGCTCAGGGTGAAAATCGTGGAAGAGAGGGCTCGGTTCGACAGAGACCCAAAGGCCTTCCGTGACTCGTACAAAGAGGAGCAGGAGAAGCTCCAGGACCAGATCTCATCCGCACGGAGTAACCTTGGTGCCGTGCAGATCGACCATGACCTCCGTGTCAAGATATCCAAGGTGTG
Sequence insert for FoMV:PDS construct (410 bp)
GAGCTGACAACAATCTATCAAGAGTTGCTTCGACATGGATACTGGCTGTTTATCATCTATGAAGATTACTGGAGTGAGCCAAGCAAGATCTTTTGCGGGAAAACTTCCTACCCAGAGATGCTTTGCAAGTAGTCACCTTGCAAGCTTTGCTGTGAAATCTCTTATCTTGAGGAATAAAGGAAGAAGCTCACACCGTAGACATTCTGCTTTGCAGATTGTCTGCAAGGATTTTCCAAGACCTCCGCTAGAAAGCACAATAAACTATTTGGAAGCTGGACAGCTCTCTTCATTTTTTAGGAACAGCGAACGCCCCAGTAAACCCTTACAGGTCGTGATTGCTGGTGCAGGATTAGCTGGTCTTTCAACAGCAAAATATCTGGCAGATGCTGGCCATAAACCCATATTGCTTG

Supplementary Table S3: Primers used for this study

Primer name	Primer sequence (5' to 3')	Function
FoMV_f	TCTGTACCGTACGATGAGCCC	Primer for RT-PCR and sequencing
FoMV_r	GCTGCGTTACTGTTAGGTCG	
Actin_f	CAGCCATCCATGATCGGTATG	Reference gene for qPCR
Actin_r	TGCCGTACAGGTCCTTTCTGA	
18srRNA_f	CTACCACATCCAAGGAAG	Reference gene for qPCR
18srRNA_r	CAATTACCAGACACTAACG	
ChID_f	CACCAGCCGAGATCAGGTTT	qPCR primer for ChID
ChID_r	AAGACGTTTCCGGGCCATT	
ChII_f	AGGGACAAGGTCACAGTGGA	qPCR primer for ChII
ChII_r	GCAAACCCGAGTCAATGGAT	
PDS_f	TGGCGAGCTTGGTATTGAGG	qPCR primer for PDS
PDS_r	TGGGAAATCAAACCGGCTGA	
PvChID_XbaI_F	GGCTCTAGACACAAGATCAACAGCAAG	Primers used for construction of the FoMV:ChID plasmid
PvChID_PacI_R	GGGTTAATTAACAGTTTTTGGTACGGTGCAG	
PvChII_XbaI_F	TGCTCTAGACCAACAGGGGGATACTCT	Primers used for construction of the FoMV:ChII plasmid
PvChII_PacI_R	GGGTTAATTAACACACCTTGGATATCTTGACAC	
PvPDS_PacI_F	GCGTTAATTAAGAGCTGACAACAATCTATCAAGAG	Primers used for construction of the FoMV:PDS plasmid
PvPDS_XbaI_R	GCCTCTAGACAAGCAATATGGGTTTATGG	

Supplementary Table S4: Phenotypic evaluations for the different developmental stages and FoMV constructs.

**Construct**	**Total number of inoculated plants**		**Plants with phenotypic change [%]**	
	Inoculated at **E1 stage**	Inoculated at **E3 stage**	Inoculated at **E1 stage**	Inoculated at **E3 stage**
WT	12	12	0	0
FoMV:ev	9	12	22.2	41.7
FoMV:ChlD	9	10	66.7	80
FoMV:ChlI	8	10	75	70
FoMV:PDS	8	10	75	80

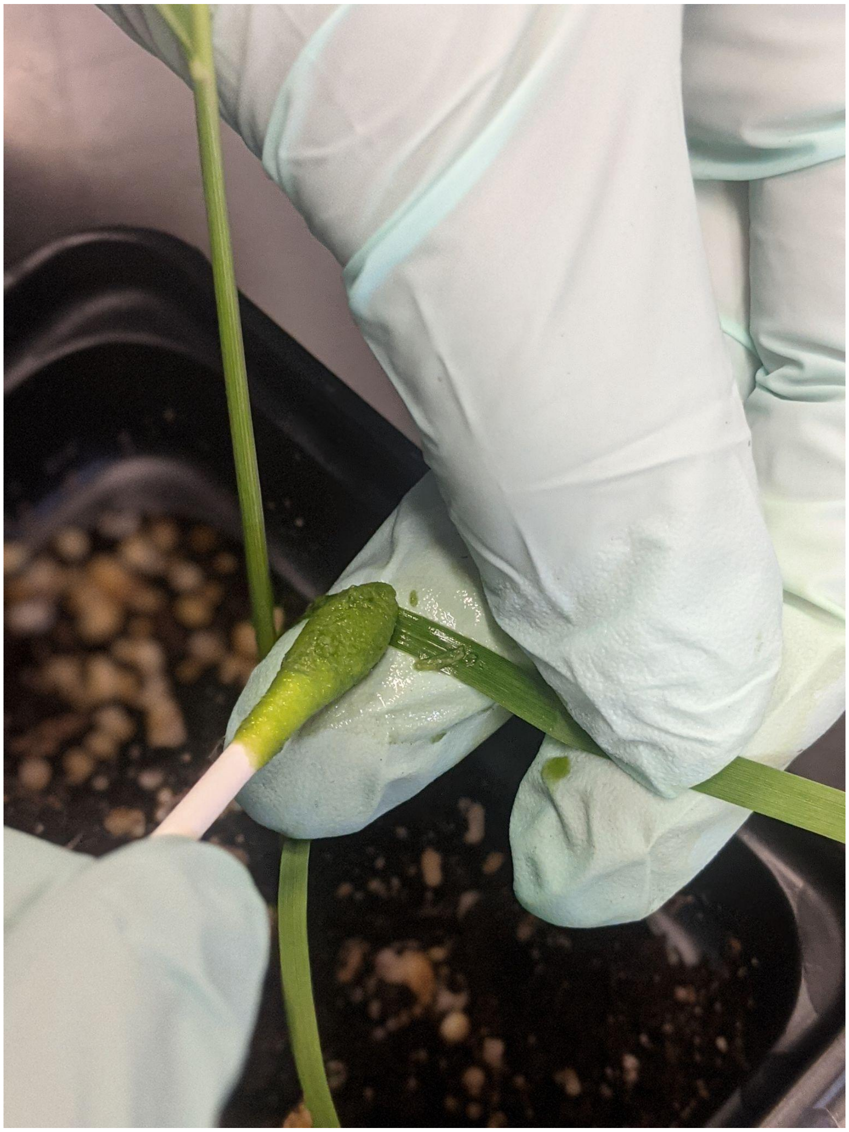

Supplementary Fig. S1: Rub-inoculation of switchgrass leaves at the elongation stage 3 (E3) with a cotton swab and the inoculum based on infected tobacco leaves.

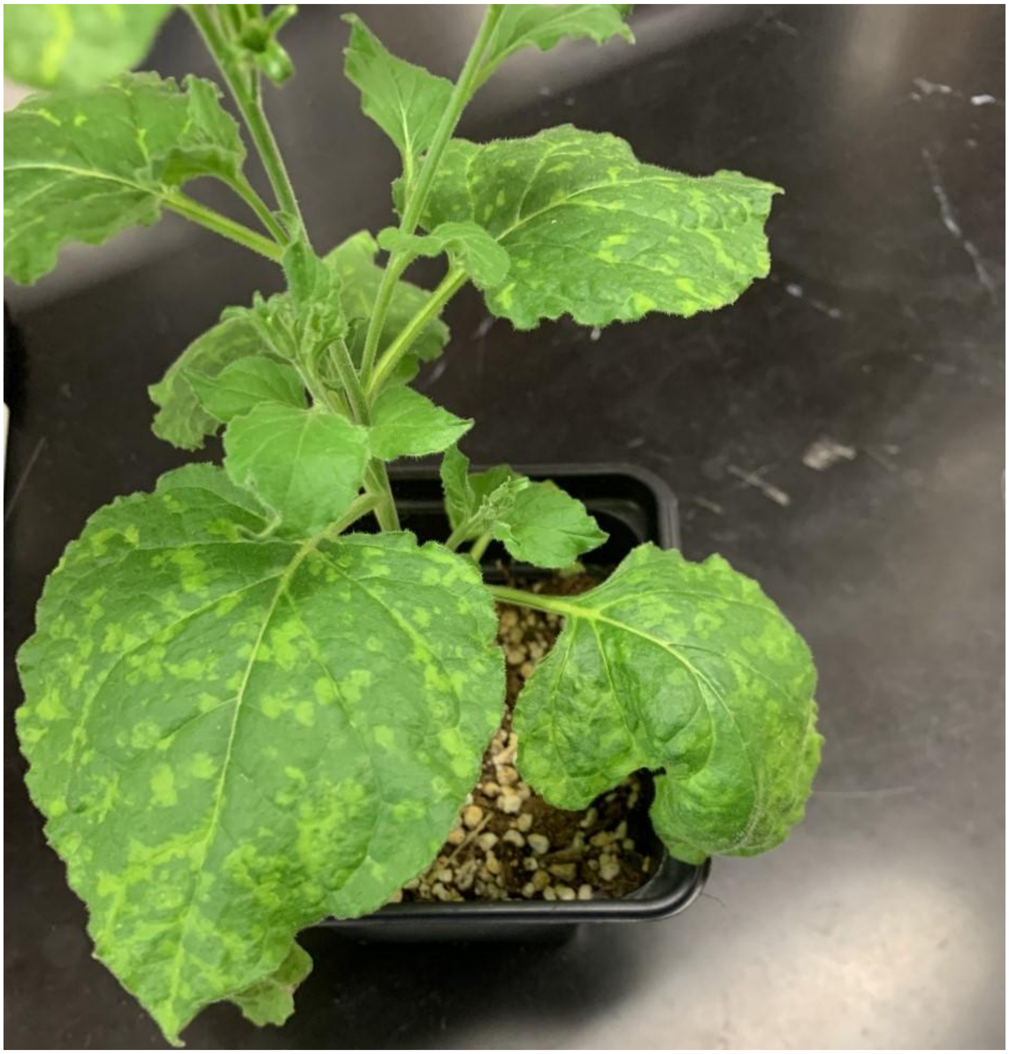

Supplementary Fig. S2: Tobacco (N. benthamiana) leaves displaying characteristic symptoms of successful infection with the Foxtail Mosaic Virus (FoMV).

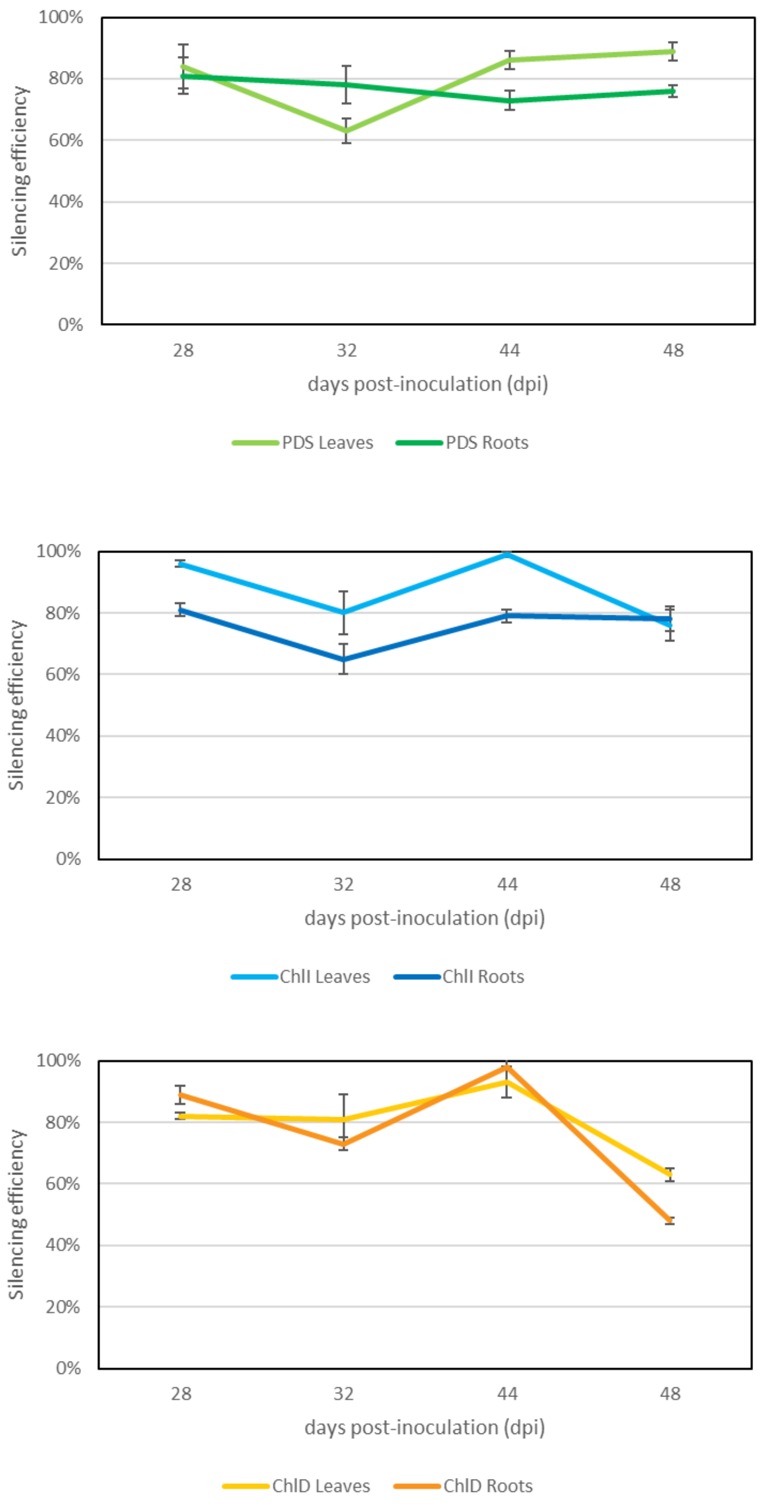

Supplementary Fig. S3: Time course experiments to test efficiency and stability of gene silencing for *PDS*, *ChlI*, and *ChlD* are following the same methods as the other experiments except for a varied period between rub-inoculation and sampling (dpi) of switchgrass plants (*n*=6).

.

```
Consensus          MGFXXTXTXXXTSLXXXXXXXXXXXXXXXXXLXSXXXRXXRLXXXXRXXAXXTXXXXJ
ChlD tobacco       MGFCSTSTLPQTSLSNSQSSTFFTYLKPCPILSSTYLRPKRLKFRLRISATAT----I
ChlD switchgrass   ---MATATALSTSLPHLPPRRVTSFPSAAVSLPS---RASRLRESRRAAAAPTASEVL

Consensus          XNGAVXXXXXXKXXXXXXXGRZYFPLAAVXGQDAIKTALLLGAIDREIGGIAIXGKRG
ChlD tobacco       PNGAVAVVEPEKQPEKISFGRQYFPLAAVIGQDAIKTALLLGAIDREIGGIAICGKRG
ChlD switchgrass   TNGAVPAA--AKSAAARGYGREYFPLAAVVGQDAIKTALLLGAIDREIGGIAISGKRG

Consensus          KTXMARGLHAXLPPIEVVVGSXANADPNXPDEWEDGLADRXZYXXDGNXKTZIVKXPF
ChlD tobacco       KTLMARGLHAILPPIEVVVGSMANADPNCPDEWEDGLADRAEYGSDGNIKTQIVKSPF
ChlD switchgrass   KTVMARGLHAMLPPIEVVVGSIANADPNEPDEWEDGLADRLQYDADGNVKTEIVKTPF

Consensus          IPLGVTEDRLIGSVDVEXSVXSGTTVFQPGLLAEAHRGVLYVDEINLLDXGISNLLLN
ChlD tobacco       IPLGVTEDRLIGSVDVEESVKSGTTVFQPGLLAEAHRGVLYVDEINLLDEGISNLLLN
ChlD switchgrass   IPLGVTEDRLIGSVDVEASVRSGTTVFQPGLLAEAHRGVLYVDEINLLDDGISNLLLN

Consensus          TEGVNIVEREGISFRHPCKPLLIATYNPEEGXVREHLLDRIAINLSADLPMSFDDRVA
ChlD tobacco       TEGVNIVEREGISFRHPCKPLLIATYNPEEGAVREHLLDRIAINLSADLPMSFDDRVA
ChlD switchgrass   TEGVNIVEREGISFRHPCKPLLIATYNPEEGSVREHLLDRIAINLSADLPMSFDDRVA

Consensus          DIATXFQEXSXEVFKMVXEETXXAKTQIILAREYLKDVXISXXQLKYLVMEAIRGGCQ
ChlD tobacco       DIATRFQECSNEVFKMVDEETDSAKTQIILAREYLKDVTISRDQLKYLVMEAIRGGCQ
ChlD switchgrass   DIATQFQESSKEVFKMVEEETEVAKTQIILAREYLKDVNISTEQLKYLVMEAIRGGCQ

Consensus          RAELYAARVAKCLAAXXGREKVXVDXLKKAVELVILPRSXJXXNPXDQQXZQPXXPPP
ChlD tobacco       RAELYAARVAKCLAAIDGREKVGVDELKKAVELVILPRSTIVENPPDQQNQQP--PPP
ChlD switchgrass   RAELYAARVAKCLAAMEGREKVFVDDLKKAVELVILPRSILADNPQDQQQEQPPQPPP

Consensus          PPZNQDSSEXQBEEEEXXZEDXEXXXXXENEQQXXQXPXEFIFDAEGGLVDXKLLFFA
ChlD tobacco       PPQNQDSSEEQNEEEEKEEEDQEDEKDRENEQQQPQVPDEFIFDAEGGLVDEKLLFFA
ChlD switchgrass   PPENQDSSEDQDEEEEDDQEDDEE----ENEQQDQQIPEEFIFDAEGGLVDDKLLFFA

Consensus          AQRRXGKAGRAKXVIFSEDRGRYIKPMLPKGPVXRLAVDATLRAAAPYQKLRXAKXXX
ChlD tobacco       AQRRKGKAGRAKKVIFSEDRGRYIKPMLPKGPVKRLAVDATLRAAAPYQKLRRAKDIQ
ChlD switchgrass   AQRRRGKAGRAKNVIFSEDRGRYIKPMLPKGPVRRLAVDATLRAAAPYQKLRKAKESD

Consensus          RKVXVEKTDMRAKRMARKAGALVIFVVDASGSMALNRMQNAKGAALKLLAESYTSRDQ
ChlD tobacco       RKVYVEKTDMRAKRMARKAGALVIFVVDASGSMALNRMQNAKGAALKLLAESYTSRDQ
ChlD switchgrass   RKVFVEKTDMRAKRMARKAGALVIFVVDASGSMALNRMQNAKGAALKLLAESYTSRDQ

Consensus          IIPFRGDXAEVLLPPSRSIXMARXRLEXLPCGGGSPLAHGLXTAVRVGXNAEKSGDVG
ChlD tobacco       IIPFRGD-AEVLLPPSRSI-MARNRLERLPCGGGSPLAHGLTTAVRVGMNAEKSGDVG
ChlD switchgrass   IIPFRGDYAEVLLPPSRSIAMARKRLEKLPCGGGSPLAHGLSTAVRVGLNAEKSGDVG

Consensus          MIVAITDGRANXSLKRSTDPEXAXASDAPRPSSXELKDEILEVAGKIYKXGMSLLVID
ChlD tobacco       MIVAITDGRANISLKRSTDPE-AEASDAPRPSSQELKDEILEVAGKIYKTGMSLLVID
ChlD switchgrass   MIVAITDGRANVSLKRSTDPEAAAASDAPRPSSKELKDEILEVAGKIYKAGMSLLVID

Consensus          NKFVSTGFAKEIARVAQGKYYYLPNASDAVISAATKXALXXLKXS
ChlD tobacco       NKFVSTGFAKEIARVAQGKYYYLPNASDAVISAATKDALSALKE-
ChlD switchgrass   NKFVSTGFAKEIARVAQGKYYYLPNASDAVISAATKTALTDLKGS
```

Supplementary Fig. S4: Alignment of the amino acid sequences of Mg-Chelatase D Subunits (ChlD) from tobacco and switchgrass (Identity = 81.86%).

```
Consensus         MPLLPTAMASXXXTXSXXXAXXXXXXXXXXXPXXXXXXXSSGXXXXXRKXXGXXXXXX
ChlI tobacco      -------MASLLGTSSSAAALASTPLSRSCKPAVFSLFPSSGQ-SQGRKFYGGIRVPV
ChlI switchgrass  MPLLPTAMASPFSTESPTSAARALLPASTSRPLSLAAAASSGRVPPSRKGLG-----F

Consensus         GRXXXXXXXXNVAXXXXXXXXXXXXXXXESQRPVYPFAAIVGQDEMKLCLLLNVIDPK
ChlI tobacco      GRSQFHVAISNVATEINLLKNRVRNLLEESQRPVYPFAAIVGQDEMKLCLLLNVIDPK
ChlI switchgrass  GR----FTVCNVAAPTAAEQEATASGAKESQRPVYPFAAIVGQDEMKLCLLLNVIDPK

Consensus         GVMIMGDRGTGKSTTVRSLVDLLPXIXVXXGDPFNSDPDXXEVMXXEVRXXXXXGXXX
ChlI tobacco      GVMIMGDRGTGKSTTVRSLVDLLPEI-VISGDPFNSDPDQ-EVMA-EVR-KLRSGQ--
ChlI switchgrass  GVMIMGDRGTGKSTTVRSLVDLLPDIRVVVGDPFNSDPDDPEVMGPEVRQRVLQGDTT

Consensus         XXXXKIXMVDLPLGATEDRVCGTIDIEKALTEGVKAFEPGLLAKANRGILYVDEVNLL
ChlI tobacco      ISRTKINMVDLPLGATEDRVCGTIDIEKALTEGVKAFEPGLLAKANRGILYVDEVNLL
ChlI switchgrass  VTTAKITMVDLPLGATEDRVCGTIDIEKALTEGVKAFEPGLLAKANRGILYVDEVNLL

Consensus         HLVDVLLDSAASGWNTVEREGISISHPARFILIGSGNPEEGELRPQLLDRFGMHAQVG
ChlI tobacco      HLVDVLLDSAASGWNTVEREGISISHPARFILIGSGNPEEGELRPQLLDRFGMHAQVG
ChlI switchgrass  HLVDVLLDSAASGWNTVEREGISISHPARFILIGSGNPEEGELRPQLLDRFGMHAQVG

Consensus         RDAELRVKIVEERARFDXBPKXFRXSYKXEQEKLQBQIXSARXXLXAVXIDHDLRVKI
ChlI tobacco      RDAELRVKIVEERARFDKNPKEFRESYKAEQEKLQNQIDSARNALSAVTIDHDLRVKI
ChlI switchgrass  RDAELRVKIVEERARFDRDPKAFRDSYKEEQEKLQDQISSARSNLGAVQIDHDLRVKI

Consensus         VCXELNVDGLRGDIVTNRAAXALAXLKGRDKVTXEDIATVIPNCLRHRLRKDPLESID
ChlI tobacco      VCAELNVDGLRGDIVTNRAARALAALKGRDKVTPEDIATVIPNCLRHRLRKDPLESID
ChlI switchgrass  VCSELNVDGLRGDIVTNRAAKALASLKGRDKVTVEDIATVIPNCLRHRLRKDPLESID

Consensus         XLVXEKFYEXFS
ChlI tobacco      VLVVEKFYEVF-
ChlI switchgrass  LLVIEKFYEIFS
```

Supplementary Fig. S5: Alignment of the amino acid sequences of Mg-Chelatase I Subunits (ChlI) from tobacco and switchgrass (Identity = 75.94%).

```
Consensus          MXXXGXXSXXXXXXXGXSXXXXXXRSXXGXXXXXXXXQRXXXCFXSSXXXXXXXXXXX
PDS tobacco        MPQIGLVSAVNLRVQGNSAYLWSSRSSLGTESQDVCLQRNLLCFGSSDSMGHKLRIRTP
PDS switchgrass    -MDTGCLSS--MKITGVS----QARSFAG----KLPTQR---CFASSHLASFAVKSLIL

Consensus          XXXRXXXXXXXXLXXVCXDXPRPXLXXTXNYLEAXXLSSXFRXSXRPXKPLZXVIAGAG
PDS tobacco        ATTRRLTKDFNPLKVVCIDYPRPELDNTVNYLEAALLSSSFRTSSRPTKPLEIVIAGAG
PDS switchgrass    NKGRSSHRRHSALQIVCKDFPRPPLESTINYLEAGQLSSFFRNSERPSKPLQVVIAGAG

Consensus          XGLSTAKYLADAGHKPILLEARDVLGGKVAAWKDXDGDWYETGLHIFFGAYPNXQNLFG
PDS tobacco        GGLSTAKYLADAGHKPILLEARDVLGGKVAAWKDDDGDWYETGLHIFFGAYPNMQNLFG
PDS switchgrass    AGLSTAKYLADAGHKPILLEARDVLGGKVAAWKDEDGDWYETGLHIFFGAYPNIQNLFG

Consensus          LGIXDRLQWKEHSMIFAMPNKPGEFSRFDFPEXLPAPXNGIXAILKNNEMLTWPEKVKF
PDS tobacco        LGIDDRLQWKEHSMIFAMPNKPGEFSRFDFPEALPAPLNGILAILKNNEMLTWPEKVKF
PDS switchgrass    LGIEDRLQWKEHSMIFAMPNKPGEFSRFDFPETLPAPVNGIWAILKNNEMLTWPEKVKF

Consensus          IGLLPAMLGGQXYVEAQDXLXVXXWMXKQGVPDRVXDEVFIAMSKALNFINPDELSMQC
PDS tobacco        IGLLPAMLGGQSYVEAQDGLSVKDWMRKQGVPDRVTDEVFIAMSKALNFINPDELSMQC
PDS switchgrass    IGLLPAMLGGQPYVEAQDVLTVSEWMKKQGVPDRVNDEVFIAMSKALNFINPDELSMQC

Consensus          LIALNRFLQEKHGSKMAFLDGNPPERLCMPIVXHIXSXGGZVRLNSRJKKIELNXDGXV
PDS tobacco        LIALNRFLQEKHGSKMAFLDGNPPERLCMPIVEHIESKGGQVRLNSRIKKIELNEDGSV
PDS switchgrass    LIALNRFLQEKHGSKMAFLDGNPPERLCMPIVDHIRSRGGEVRLNSRLKKIELNPDGTV

Consensus          XFXLXBGXXIXGDAXVXAXPVDIXKLLXPZXWXEIXYFXKLEKLVGVPVINVHIWFDRK
PDS tobacco        CFILNNGSTIKGDAFVFATPVDILKLLLPEDWKEIPYFQKLEKLVGVPVINVHIWFDRK
PDS switchgrass    YFALTDGTQITGDAYVCAAPVDIFKLLVPQEWSEISYFKKLEKLVGVPVINVHIWFDRK

Consensus          KNTXDXLLFSRSXLLSVYADMSVTCKEYYBPNXSMLELVFAPAEEWIXRSDXEIIDATM
PDS tobacco        KNTSDNLLFSRSPLLSVYADMSVTCKEYYNPNQSMLELVFAPAEEWINRSDSEIIDATM
PDS switchgrass    KNTYDHLLFSRSSLLSVYADMSVTCKEYYDPNRSMLELVFAPAEEWIGRSDAEIIDATM

Consensus          ELAKLFPDEIXADXSKAKILKYHVVKTPRSVYKTVPXCEPCRPLQRSPIEGFYLAGDYT
PDS tobacco        ELAKLFPDEISADQSKAKILKYHVVKTPRSVYKTVPGCEPCRPLQRSPIEGFYLAGDYT
PDS switchgrass    ELAKLFPDEIAADLSKAKILKYHVVKTPRSVYKTVPNCEPCRPLQRSPIEGFYLAGDYT

Consensus          QKYLASMEGAVLSGKLCAQXIVQDYELLLGRSQKMLAEASVVSIVN
PDS tobacco        QKYLASMEGAVLSGKLCAQAIVQDYELLLGRSQKMLAEASVVSIVN
PDS switchgrass    QKYLASMEGAVLSGKLCAQSIVQ-----------------------
```

Supplementary Fig. S6: Alignment of the amino acid sequences of phytoene desaturase (PDS) from tobacco and switchgrass (Identity = 78.11%).

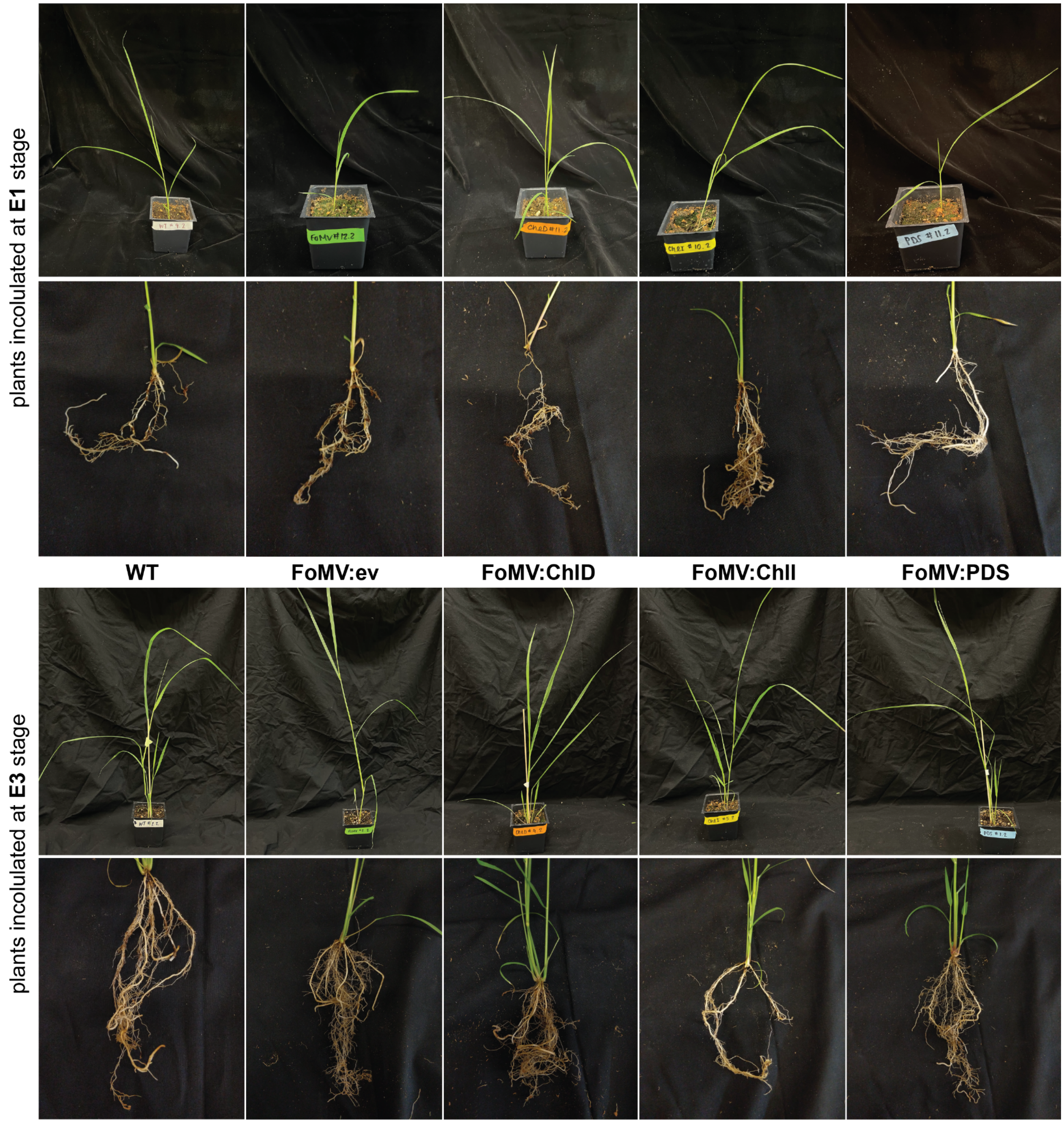

Supplementary Fig. S7: Photos of switchgrass plants that were infected at different developmental stages (either E1 or E3).
